# Factors associated with poor treatment outcome of tuberculosis in Debre Tabor, northwest Ethiopia

**DOI:** 10.1186/s13104-018-3129-8

**Published:** 2018-01-15

**Authors:** Addisu Melese, Balew Zeleke

**Affiliations:** 1 Department of Medical Laboratory Science, College of Health Sciences, Debre Tabor University, P.O.BOX 272, Debre Tabor, Ethiopia; 2 Department of Nursing, College of Medicine and Health Science, Bahir Dar University, Bahir Dar, Ethiopia

**Keywords:** Tuberculosis, Treatment outcome, Debre Tabor, Ethiopia

## Abstract

**Objective:**

Directly observed treatment short course has been implemented as part of the national tuberculosis control program in Ethiopia. The strategy, as evidenced by different studies, has improved the survival and treatment success rate of tuberculosis patients. However, some patients failed to complete their treatments and the factors for this failure were not assessed in the study area. We, therefore sought to identify factors associated with poor treatment outcome of tuberculosis in Debre Tabor, northwest Ethiopia.

**Results:**

We included 303 patients (173 males, 130 females) with mean age of 34.9 years in the study and 39 (12.9%) patients were with poor treatment outcome over the period of 5 years (2008–2013). Being male, urban residency, positive and unknown smear result at the 2nd month of treatment and patients in the age of 35–44 years were more likely to have poor treatment outcomes than their counterparts. Patients in the new treatment category were less likely to have poor treatment outcome compared to the retreated cases. Further studies are recommended to explore the association of poor treatment outcome with other important factors which are not investigated by this study.

**Electronic supplementary material:**

The online version of this article (10.1186/s13104-018-3129-8) contains supplementary material, which is available to authorized users.

## Introduction

Despite intensive efforts to control, tuberculosis remains a major public health problem worldwide. The problem is further burdened with the spread of HIV/AIDS [1]. In 2015, 10.4 million people were newly infected with TB and 1.4 million deaths were reported. Of these, HIV patients accounted 1.2 millions of all new cases and 0.4 million of additional deaths. In the same year, 0.48 million new multi-drug resistant TB (MDR-TB) were reported [2]. With the aim of detecting 70% of cases and curing 85% of sputum positive patients under treatment, World Health Organization (WHO) launched directly observed treatment short course (DOTS) [3] and saved more than 49 million lives through effective diagnosis and treatment between 2000 and 2015 [2].

Ethiopia has adopted DOTS as a strategy in its national tuberculosis control program. The strategy, as evidenced by the government and studies conducted in the country, has improved the survival [4] and treatment success rate of tuberculosis patients [4–13]. For effectiveness of the strategy, Ethiopia provided free diagnosis and treatment both in public and private health facilities through public–private mix (PPM) approach that was found effective in expanding DOTS to increase case detection and treatment success rates [14].

Although medication for TB treatment is available for free in Ethiopia, many patients were found with poor treatment outcome (failures, defaulters, deaths) [15]. This poor treatment may lead to pronged period of infection, drug resistance, increased morbidity and mortality. Failure to complete treatments indicate the presence of poor treatment outcome [16]. Different studies revealed that various factors are associated with poor treatment outcome including pulmonary TB [5, 13], HIV co-infection [5, 13, 16, 18], re-treatment [13, 18], smear negativity at initiation of treatment [15, 18], sputum smear positivity at 2nd month of treatment [15, 18], being male [18], younger and older patients [18], drug resistance [21], and living in rural area [23]. Although studies are being conducted on treatment outcomes, factors associated with poor treatment outcomes were not assessed in the study area. Therefore; this study was aimed to identify factors associated with poor treatment outcome among TB patients in Debre Tabor, northwest Ethiopia.

## Main text

### Study design and setting

A retrospective study was conducted at Debre Tabor health center, DOTS clinic in Debre Tabor. Patients were followed until the end of treatment to determine their treatment outcomes in accordance with the national TB and leprosy control program guideline (NTLCP) [17].

### Study participants

All patients registered for TB treatment at Debre Tabor health center from May 2008–April 2013 and had known treatment outcome were included in the analysis. Patients with unknown treatment outcomes were excluded from the analysis. Over the 5 years, a total of 339 TB patients started their treatments at the health center but only 303 patients (89.4%) of the total registered patients were eligible and included in the study while 36 patients transferred to other facilities.

### Data collection

Socio-demographic and clinical data of patients were taken from DOTS clinic of Debre Tabor health center. Medical records were reviewed and collected by nurses using data sheet designed for this study (Additional file 1). Age, sex, resident, type of TB, category of patients, HIV status, smear result, treatment year and treatment outcomes were the extracted from the registers.

### Data analysis

Data were entered and analyzed using SPSS 20.0 and described using frequencies, proportions, 95% CI crude and adjusted odds ratios (OR). A two sided statistics was used during the analysis. Logistic regressions and P values < 0.05 were used to identify factors associated with poor treatment outcomes.

### Results

A total of 303 patients with mean age of 34.9 years were included in the study. Majority of them were males (57.1%) and 61% were living in the urban. During diagnosis, almost three-fourth (73.3%) were smear negative TB patients. Based on this; 26.7% were smear positive PTB, 34.7% were smear negative PTB and 38.6% were extra pulmonary TB. Drug resistance for smear positive patients was done through referral system and only one patient was found with MDR TB and transferred to MDR treatment center as there was no treatment provided for MDR TB at the clinic. At the start of treatment, 88.1% of patients were new cases, 5.0% were re-treated, 5.6% were transferred in and 1.3% were others. Sputum smear tests were done at the 2nd month for 25.4%, at 5th month for 24.8% and at 7th month for 23.1% of patients. After 6–8 months of treatment, 39 (12.9%) patients had poor treatment outcome of which, 12 failed, 8 defaulted and 19 died.

Among the 39 patients with poor treatment outcome, 12 (30.8%) were failures, 8 (20.5%) were defaulters and 19 (48.72%) were deaths. Majority of treatment failures, defaults, and deaths were observed among males and urban residents. Of those with treatment failure, higher rate (33.3%) was observed in the age of 45–54 years. Among defaulters, higher rates (37.5%) were registered among patients in the age of 15–24 and ≥ 65 years while high rates (31.6%) of deaths were reported for patients ≥ 65 years. HIV negative patients were more likely to have treatment failure and patients with unknown HIV result had higher rates of deaths.

Majority of patients with poor treatment outcome (38.5%) were having extrapulmonary TB. Among smear positive PTB patients, 16.05% had poor treatment outcome. Smear positive PTB patients had higher rate of treatment failure than smear negative PTB and extra pulmonary PTB. Higher rates of default was observed among extrapulmonary while higher rates of deaths were seen among smear negative PTB patients. Majority (71.8%) of patients with poor treatment outcome had unknown smear result at the 2nd months of treatment (either not done or results were not recorded) (Table 1).

**Table 1:** Socio-demographic and clinical profile of TB patients and characteristics of patients with poor treatment outcomes at Debre Tabor health center, N = 303

Variables	Total N (%)	Poor treatment outcome			
		Failed	Defaulted	Died	Total
Sex					
Male	173 (57.1)	9 (75.0)	6 (75.0)	14 (73.7)	29 (74.4)
Female	130 (42.9)	3 (25.0)	2 (25.0)	5 (26.3)	10 (25.6)
Residence					
Urban	187 (61.7)	8 (66.7)	6 (75.0)	15 (78.9)	29 (74.4)
Rural	116 (38.3)	4 (33.3)	2 (25.0)	4 (21.1)	10 (25.6)
Age group					
≤ 14	20 (6.6)	1 (8.3)	0 (0.00)	0 (0.0)	1 (2.6)
15–24	68 (22.4)	3 (25.0)	3 (37.5)	1 (5.3)	7 (17.9)
25–34	83 (27.4)	2 (16.7)	1 (12.5)	3 (15.8)	6 (15.4)
35–44	47 (15.5)	1 (8.3)	1 (12.5)	4 (21.1)	6 (15.4)
45–54	31 (10.2)	4 (33.3)	0 (0.00)	2 (10.5)	6 (15.4)
55–64	25 (8.3)	0 (0.00)	0 (0.00)	3 (15.8)	3 (7.7)
≥ 65	29 (9.6)	1 (8.3)	3 (37.5)	6 (31.6)	10 (25.6)
Baseline smear result					
Positive	81 (26.73)	10 (83.3)	1 (12.5)	2 (10.5)	13 (33.3)
Negative	222 (73.27)	2 (16.7)	7 (87.5)	17 (89.5)	26 (66.7)
Type of TB					
Smear positive PTB	81 (26.7)	10 (83.3)	1 (12.5)	2 (10.5)	13 (33.3)
Smear negative PTB	105 (34.7)	1 (8.3)	0 (0.00)	10 (52.6)	11 (28.2)
Extra pulmonary TB	117 (38.6)	1 (8.3)	7 (87.5)	7 (36.8)	15 (38.5)
Smear result at the 2nd month					
Positive	10 (3.3)	7 (58.3)	0 (0.00)	0 (0.00)	7 (17.9)
Negative	67 (22.1)	3 (25.0)	1 (12.5)	0 (0.00)	4 (10.3)
Unknown	226 (74.6)	2 (16.7)	7 (87.5)	19 (100)	28 (71.8)
HIV status					
Positive	41 (13.5)	2 (16.7)	2 (25.0)	2 (10.5)	6 (15.4)
Negative	179 (59.1)	9 (75.0)	3 (37.5)	7 (36.8)	19 (49.7)
Unknown	83 (27.4)	1 (8.3)	3 (37.5)	10 (52.6)	14 (35.9)
Patient category					
New	267 (88.1)	8 (66.7)	8 (100.0)	17 (89.5)	33 (84.6)
Retreated	15 (5.0)	4 (33.3)	0 (0.00)	2 (10.5)	6 (15.6)
Transferred in	17 (5.6)	0 (0.00)	0 (0.00)	0 (0.00)	0 (0.00)
Unknown	4 (1.3)	0 (0.00)	0 (0.00)	0 (0.00)	0 (0.00)
Year of treatment					
May 2008–April 2009	66 (21.8)	0 (0.0)	2 (25.0)	9 (47.4)	11 (28.2)
May 2009–April 2010	68 (22.4)	5 (41.7)	4 (50.0)	4 (21.1)	13 (33.3)
May 2010–April 2011	49 (16.2)	2 (16.7)	2 (25.0)	2 (10.5)	6 (15.4)
May 2011–April 2012	59 (19.5)	1 (8.3)	0 (0.00)	4 (21.1)	5 (12.8)
May 2012–April 2013	61 (20.1)	4 (33.3)	0 (0.00)	0 (0.00)	4 (10.3)

### Factors associated with poor treatment outcome

Factors having p < 0.25 in the bivariate analysis were entered to the multivariate analysis. In the multivariate analyses, being male (AOR 2.516, 95% CI 1.101–4.816), living in urban areas (AOR 3.214 , 95% CI 1.201–8.598), smear positive result at the 2nd month of treatment (AOR 40.132, 95% CI 5.004–321.839), unknown smear result at the 2nd month of treatment (AOR 14.495, 95% CI 2.409–87.236), patients in the age of 25–34 (AOR 6.395, 95% CI 1.741–23.492), 35–44 years (AOR 15.157, 95% CI 3.495–65.743), 45–54 years (AOR 7.576, 95% CI 1.791–32.052), 55–64 years (AOR 4.850, 95% CI 1.099–21.394) and older patients in the age of above 65 years (AOR 7.291, 95% CI 1.409–37.735) were independent factors associated with poor treatment outcome. On the other hand, patients in the new treatment category were less likely to have poor treatment outcome compared to the retreated cases (AOR 0.168, 95% CI 0.035–0.798) (Table 2).

**Table 2:** Factors associated with poor treatment outcome in Debre Tabor, May 2008–April 2013; N = 303

Variable	Treatment outcome		COR (95% CI)	P value	AOR (95% CI)	P value
	Poor (%)	Successful (%)				
Sex						
Male	29 (74.4)	144 (54.6)	2.417 (1.132–5.160)	0.023	2.516 (1.101–4.816)*	0.029
Female	10 (25.6)	120 (45.4)	1		1	
Age group						
≤ 14	1 (2.6)	19 (7.2)	1	1	1	
15–24	7 (18.0)	61 (23.1)	10.0 (1.163–85.998)	0.036	8.247 (0.723–94.052)	0.089
25–34	6 (15.4)	77 (29.2)	4.586 (1.535–13.704)	0.006	6.395 (1.741–23.492)*	0.005
35–44	6 (15.4)	41 (15.5)	6.754 (2.182–20.905)	0.001	15.157 (3.495–65.743)*	0.000
45–54	6 (15.4	25 (9.5)	3.596 (1.140–11.347)	0.029	7.576 (1.791–32.052)*	0.006
55–64	3 (7.7)	22 (8.3)	2.193 (0.677–7.100)	0.190	4.850 (1.099–21.394)*	0.037
≥ 65	10 (25.6)	19 (7.2)	3.86 (0.925–16.109)	0.064	7.291 (1.409–37.735)*	0.018
Residence						
Urban	29 (74.4)	158 (59.8)	1.946 (0.91–4.159)	0.086	3.214 (1.201–8.598)*	0.020
Rural	10 (25.6)	106 (40.2)	1		1	
Baseline smear result						
Smear positive	13 (33.3)	68 (25.8)	1			
Smear negative	26 (66.7)	196 (74.2)	1.441 (0.701–2.96)	0.320		
Smear result at 2nd month						
Negative	4 (10.3)	63 (23.9)	1		1	
Positive	7 (17.9)	3 (1.1)	36.75 (6.792–198.842)	0.000	40.132 (5.004–321.839)*	0.001
Unknown	28 (71.8)	198 (75.0)	16.50 (4.032–67.530)	0.000	14.495 (2.409–87.236)*	0.004
Type of TB						
Smear positive PTB	13 (33.3)	68 (25.8)	1			
Smear negative PTB	11 (28.2)	94 (35.6)	0.769 (0.344–1.718)	0.522		
Extrapulmonary TB	15 (38.5)	102 (38.6)	1.257 (0.55–2.873)	0.588		
HIV status						
Negative	19 (48.7)	160 (60.6)	1			
Positive	6 (15.4)	35 (13.3)	1.184 (0.419–3.346)	0.751	1.360 (0.208–8.910)	0.748
Unknown	14 (35.9)	69 (26.1)	1.709 (0.81–3.602)	0.159	2.137 (0.338–11.394)	0.387
Patient category						
New	33 (84.6)	234 (88.6)	0.212 (0.071–0.633)	0.005	0.168 (0.035–0.798)*	0.025
Retreated	6 (15.4)	9 (3.4)	1		1	
Transferred in	0 (00.0)	17 (6.4)	–			
Unknown	0 (00.0)	4 (1.5)	–			
Year of treatment						
May 2008–April 2009	11 (28.2)	55 (20.8)	0.846 (0.349–2.052)	0.712	0.707 (0.140–3.578)	0.675
May 2009–April 2010	13 (33.3)	55 (20.8)	1.433 (0.491–4.186)	0.510	0.616 (0.077–4.951)	0.649
May 2010–April 2011	6 (15.4)	43 (16.3)	2.160 (0.703–6.632)	0.178	1.228 (0.144–10.500)	0.851
May 2011–April 2012	5 (12.8)	54 (20.5)	2.850 (0.856–9.489)	0.088	2.453 (0.269–22.385)	0.426
May 2012–April 2013	4 (10.3)	57 (21.6)	1		1	

*Significant at P < 0.05

### Discussion

Identifying factors associated with poor treatment outcome could help to evaluate the performance of TB control programs and to design effective interventions. Although DOTS programs invariably reported treatment outcomes for smear positive PTB, it was recently smear negative PTB and EPTB got a concern. Hence; studies conducted across different populations identify various factors that could affect treatment outcomes of all cases of TB [5, 10–13, 15, 16, 18–24].

This study revealed overall poor treatment outcome of 12.9% which was relatively higher than a report from Tigray with a rate of 10.8% [10]. In contrast, this outcome was lower than a study conducted in southern Ethiopia (16.7%) [18], (14.8%) [23] and Georgia (19.7%) [19]. The lower poor outcome in our study could be explained in terms of patients population, study period and the relatively higher rates of transferred out patients (10.62%) that could have poor outcome. Because there was no a system of tracing outcomes of these patients, they were excluded from the analysis since their final outcome was not known.

Among patients with poor treatment outcome; majority (41.72%) had death, 30.8% had failure and 20.5% had default outcome. In contrast, previous studies reported that majority of the patients with poor outcome were defaulted followed by deaths in southern region of Ethiopia (61, 36%) [18] and southern Ethiopia (11.1, 3.4%) [23]; respectively. This could be due to misreporting of defaulted patients as dead since this study could not trace who was actually dead, defaulted and failed. In all aspects of poor treatment outcome, higher rates were seen among new TB cases. These new cases were more likely to die than to fail and default while re-treated cases were more likely to fail than to default and die. Although retreatment cases were more likely to fail and supported by studies in southern Ethiopia, higher failure rates were reported in retreatments than new cases [18]. This discrepancy might be due to improper categorization of patients during deciding the final treatment outcomes in our study.

In this study, patients living in urban areas had poor treatment outcome than rural residents (AOR: 3.214). Although not significant, this finding is supported by reports from southern Ethiopia [18]. The relatively higher co-morbidities (including HIV, diabetes mellitus), temporary interruption of treatment and other addiction related factors among urban residents might explain this difference. Contrary to our findings, studies from central and southern Ethiopia [22, 23] reported that rural residents are more likely to have poor treatment outcome due to poor follow up [25], poor awareness about the disease and its treatment, longer distances to treatment center and associated costs [20, 21, 24, 26] may force them to interrupt and discontinue their treatments. Further prospective studies might be needed to solve such discrepancies.

A follow up smear was done at the 2nd, 5th and 7th months of treatment. Patients with smear positive result at the 2nd month of treatment had higher poor outcome compared to smear negative result (AOR: 40.132). In addition, smear positive patients at the 2nd month of treatment were almost three times more likely to have poor treatment outcome than patients with unknown smear result at the 2nd month of treatment. Patient attitude and behavior towards the disease [4, 18, 20] could be the possible explanation in that subsidence of symptoms after completing the initiation phase may lead them to feel better and assume they are cured or patients may loss interest in continuing treatment if symptoms persist and no improvements are seen. This could result patients to default, to fail or to die and hence poor outcome.

In this study, males had poor treatment outcome than females (AOR: 2.516). Other studies conducted from South Ethiopia [4] reported similar scenario and this difference might be related with biological, educational, social, economical, behavioral and environmental factors which are not investigated in this study. On the other hand, studies from Georgia and Egypt reported that females are more likely to have poor treatment outcome than males due to poor health seeking behavior and poor health access for females than males. In addition, low monthly income, unemployment and uneducated patients were reported as potential risk factor for poor outcome [19, 27] that could work either of the two.

Patients in the age of 25–34 years old had almost 2.5 times (AOR: 15.157) poor treatment outcome than in the age groups of 15–24 years (AOR: 6.395), two times than 34–44 years (AOR: 7.576), three times than 45–54 years (AOR: 4.85) and two times than 55–64 years (AOR: 7.291) compared to the above 65 years. Studies from southern Ethiopia supported that the advanced age population is the most affected and with poor treatment outcome [23, 25, 26]. Literatures reported drug resistance [21], poor adherence to treatment [13, 20, 21] and HIV coinfection [5, 15, 16, 18] as major factors leading to poor treatment outcome. Although not significant; baseline smear positive result, unknown and HIV positive status, EPTB patients and treatment in the year May 2011–April 2012 had poor treatment outcome than their counterparts.

## Conclusions

Factors associated with poor treatment outcome can be used to identify patients who could have poor outcome during treatment. Males, urban residency, positive and unknown smear result at the 2nd month of treatment and patients in the age of 35–44 years were more likely to have poor treatment outcome during treatment than their counterparts. Considerable numbers of patient information were missed or unknown and patient registers should be improved and synchronized with important patients’ data. Further studies are recommended to explore the association of poor treatment outcome with other important factors which are not investigated by this study.

## Limitations

- The retrospective nature of the study design fails to identify potential factors that could affect treatment outcome including patient attitude towards the disease, education, occupation, nutrition, distance from treatment center, monthly income and others.
- It did not trace out the treatment outcome of transferred out and defaulted patients.

## Additional file

**Additional file 1.** Data collecting tool for the assessment of treatment outcome of tuberculosis in Debre Tabor, Northwest Ethiopia.

## Abbreviations

TB: tuberculosis
PTB: pulmonary tuberculosis
EPTB: extra-pulmonary tuberculosis
DTHC: Debre Tabor health center
NTLCP: National Tuberculosis and Leprosy Control Program
DOTS: directly observed treatment short course
PPM: public–private mix
