# Correction to: Factors associated with poor treatment outcome of tuberculosis in Debre Tabor, northwest Ethiopia

**DOI:** 10.1186/s13104-020-05062-w

**Published:** 2020-05-07

**Authors:** Addisu Melese, Balew Zeleke

**Affiliations:** 1 Department of Medical Laboratory Science, College of Health Sciences, Debre Tabor University, P.O.BOX 272, Debre Tabor, Ethiopia; 2 Department of Nursing, College of Medicine and Health Science, Bahir Dar University, Bahir Dar, Ethiopia

## Correction to: BMC Res Notes (2018) 11:25 10.1186/s13104-018-3129-8

Following publication of the original article [1], similarity with another article by the same authors was reported. The authors would like to clarify that the current study [1] is an update of previous work that used the same data set in the analysis [2]. In Melese and Zeleke [1], the authors found that factors associated with poor treatment outcome were not sufficiently addressed previously. As a result of the reanalysis the effective sample size has changed.
