# Supplementary material for: Factors associated with poor treatment outcome of tuberculosis in Debre Tabor, northwest Ethiopia
Source: BMC Res Notes. 2018 Jan 15;11:25. doi: 10.1186/s13104-018-3129-8 (PMC5769532; doi:10.1186/s13104-018-3129-8)
Supplement: Supplementary file 1 — Additional file 1. Data collecting tool for the assessment of treatment outcome of tuberculosis in Debre Tabor, Northwest Ethiopia. [file 13104_2018_3129_MOESM1_ESM.docx]

**Data collecting tool for the assessment of treatment outcome of tuberculosis in Debre Tabor**

**Code:____________**

| **S.no** | **Variable** | **Responses** |
| --- | --- | --- |
|  | Treatment center | _______________ |
|  | Year of treatment | 1. May 2008-April 2009 2. May 2009-April 2010 3. May 2010-April 2011 4. May 2011-April 2012 5. May 2012-April 2013 |
|  | Sex of Patient | 1. Male 2. Female |
|  | Age | ______ (in years) |
|  | Residence: | 1. Urban 2. Rural |
|  | Type of TB | 1. Smear +ve pulmonary 2. Smear -ve Pulmonary 3. Extra PTB |
|  | Patient category | 1. New 2. Relapse 3. Failure 4. Default 5. Transfer in 6. Unknown |
|  | Smear result | 1. Base line: Positive ______Negative _______ 2. 2^nd^ month: Positive ____, Negative _____Not done ______ 3. 5^th^ month: Positive _____, Negative _____Not done ______ 4. 7^th^ month: Positive ____, Negative _______ Not done _____ |
|  | HIV status | 1. HIV +Ve 2. HIV –Ve 3. Not done |
|  | Treatment outcome | 1. Cured 2. Completed 3. Failure 4. Defaulted 5. Died 6. Transferred out 7. Unknown |

Name of data collector: _______________________________ Sign: _________ Date: ----/---/--
